# Marsdenia tenacissima extract prevents the malignant progression of glioma through upregulating lncRNA MEG3 and SFRP1‐dependent inhibition of Wnt/β‐catenin pathway

**DOI:** 10.1111/cns.14100

**Published:** 2023-02-08

**Authors:** Lei Chen, Xin Gong, Mengyi Huang

**Affiliations:** ^1^ Department of Neurosurgery, Hunan Provincial People's Hospital The First Affiliated Hospital of Hunan Normal University Changsha Hunan China

**Keywords:** competing endogenous RNA, glioma, LncRNA MEG3, Marsdenia tenacissima extract, miR‐542‐3p, SFRP1

## Abstract

**Background/Aim:**

Recent studies have highlighted the tumor‐suppressive effect of Marsdenia tenacissima extract (MTE) on human cancers. This research unveils the potential impact of MTE on glioma and ascertains the relevant molecular mechanisms.

**Methods:**

Glioma cells were treated with MTE, with normal human astrocytes (NHAs) as controls. A battery of function experiments, including the CCK‐8 viability test, colony formation assay, scratch migration assay, and Transwell invasion assay, was executed to address the responses of glioma cells to MTE treatment and gain or loss of function of lncMEG3, miR‐542‐3p, and SFRP1. FISH, RIP, and dual‐luciferase reporter assays were adopted for assessing gene interactions. U251‐GFP‐Luc cells were delivered into nude mice through intracranial injection to develop an orthotopic glioma model for in vivo validation.

**Results:**

200 mg/mL MTE could suppress the proliferating, migrating, and invading properties of glioma cells but not affect those of NHAs. MTE treatment enhanced the expression of lncMEG3, which competes with SFRP1 for binding miR‐542‐3p. SFRP1 could inactivate the Wnt/β‐catenin pathway. Animal experimentation substantiated the antitumor activity and mechanism of MTE in nude mice.

**Conclusions:**

MTE suppresses glioma via the lncMEG3/miR‐542‐3p/SFRP1/Wnt/β‐catenin axis. These findings contribute to a theoretical basis for the use of MTE for glioma patients.

## Graphical abstract

Marsdenia tenacissima extract increases lncMEG3 expression to regulate miR‐542‐3p/SFRP1 and inhibit Wnt/β‐catenin signaling pathway, thereby suppressing glioma cell proliferation, migration, and invasion.

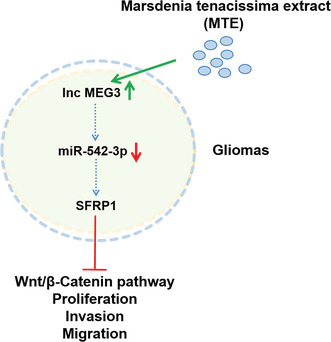

## 1 INTRODUCTION

Glioma is a brain malignancy originated from neuroglial stem/progenitor cells, accountable for the majority of deaths pertaining to primary intracranial tumors.^1^ Gliomas constitute highly heterogeneous primary tumors in the central nervous system.^2^ Although modern advancements in surgical treatment for glioma have achieved maximal cytoreduction and minimal morbidity,^3^ challenges remain to be addressed, including techniques overcoming the substantial spatial and temporal heterogeneity in the changes of molecules in gliomas and effective medicines penetrating the blood–brain barrier.^4^ Traditional Chinese medicine (TCM) has attracted great attention as an alternative therapeutic strategy against human cancers,^5^, ^6^ including glioblastoma (GBM).^7^

Marsdenia tenacissima has been defined to be a promising antitumor TCM herb.^8^ Marsdenia tenacissima extract (MTE) shows an in vitro strong antilymphoma potential, which is potentially related to its antiangiogenic potential in tumors.^9^ Furthermore, a prior study has highlighted the proapoptotic effects of ethanolic MTE on hematologic neoplasm cells, contributing to its tumor‐suppressive impact on hematological malignancy.^10^ More recent studies have uncovered the antitumor effect of MTE on human ovarian cancer^11^ and lung cancer.^12^ However, its functional significance and mechanism of action in glioma are largely undetermined.

Long noncoding RNAs (lncRNAs) are noncoding transcripts with their length surpassing 200 nucleotides, which are involved in malignant transformation.^13^ LncRNA MEG3 has shown low expression in glioma cells as well as antiproliferative and antimigratory impacts on glioma cells through modulation of a miRNA‐mRNA interaction (miR‐6088/SMARCB1).^14^ Since our in silico analysis by starBase database showed a binding relationship between lncMEG3 and miR‐542‐3p, we speculated whether lncMEG3 could mediate miR‐542‐3p to affect the progression of glioma. A latest study has documented that miR‐542‐3p is abundantly expressed in high‐grade gliomas relative to low‐grade ones and that its high expression correlates with worse outcomes.^15^ Recently, miR‐542‐3p has been suggested to target secreted frizzled‐related protein 1 (SFRP1) via pairing to its 3′UTR.^16^

SFRP1 belongs to the group of SFRPs and acts as a crucial antagonist of the canonical Wnt pathway.^17^, ^18^ Their interaction has been extensively illustrated to be mediated by miRNAs to exert regulatory roles in the progression of tumors. For instance, miR‐27a‐induced inhibition of SFRP1 can induce the Wnt/β‐catenin pathway activation, resulting in enhanced proliferation and invasiveness of osteosarcoma cells.^19^ Additionally, miR‐1301‐3p‐dependent inhibition of SFRP1 activates the Wnt pathway to attenuate the prostate cancer stem cell expansion.^20^ Furthermore, SFRP1 exerts an antitumor role in glioma through suppressing the Wnt/β‐catenin pathway.^21^

Following the aforementioned findings, we surmised that the lncMEG3/miR‐542‐3p/SFRP1/Wnt axis plays a modulatory effect of MTE in glioma. Here, both in vitro and in vivo models were utilized to testify this assumption to offer a basis for the in‐depth mechanism underlying the TCM treatments of glioma.

## 2 METHODS AND MATERIALS

### 2.1 Cell culture

A human glioma cell line U87 was available from the American Type Culture Collection (Manassas, Virginia, USA), a human glioma cell line U251 from the Cell Bank, Shanghai Institute of Biochemistry and Cell Biology, Chinese Academy of Sciences (Shanghai, China), and normal human astrocytes (NHAs) from the Cell Bank of the Chinese Academy of Sciences (Shanghai, China). U87 and U251 were cultured in Dulbecco's modified Eagle's medium (DMEM, Gibco) with 1% penicillin/streptomycin and 10% fetal bovine serum (FBS), and NHAs in an astrocyte culture medium (Life Technologies) with 1% penicillin/streptomycin and 10% FBS. Cells were cultured under the condition of 37°C and 5% CO_2_.

MiR‐542‐3p Agomir, Agomir negative control (NC), miR‐542‐3p Antagomir, Antagomir NC, lncMEG3 knockdown vector (sh‐MEG3), SFRP1 overexpression vector (pcDNA3.1‐SFRP1), NC pcDNA3.1, and NC shRNA were available from GenePharma (Shanghai, China). Transfection was subjected to the requirements of Lipofectamine 2000 reagent (Invitrogen), and the subsequent experiments were carried out 48 h later.

### 2.2 Treatment of MTE

The MTE was extracted referring to a previous study.^12^ In short, the powder of the stem of *M. tenacissima* (1 kg) was immersed into water for three times (1.5, 1, and 0.8 h) for lixiviation. Next, the extracts were pooled, filtered, and concentrated, followed by precipitation with 8 times of 85% ethanol (v/w) at 4°C for 24 h. The ethanol in the extract was recovered and 85% ethanol was supplemented again for further precipitation. Subsequently, the ethanol was recovered thoroughly, and then the insoluble precipitate was removed by filtration. Lastly, the extract was concentrated to 200 mL, diluted with water for injection, and supplemented with 0.3% polysorbate 80 (pH: 5.5–6.0). The MTE extract was identified to possess more than 95% of purity.

### 2.3 Quantitative reverse transcription‐polymerase chain reaction (qRT‐PCR)

TRIzol reagent (Invitrogen) was implemented for the extraction of total RNA, and the reverse transcription was employed with a reverse transcription kit (TaKaRa). Expression levels of genes were detected using a fluorescence qPCR instrument (LightCycler 480, Roche). The reaction conditions of PCR were implemented with the fluorescence qPCR kit (SYBR Green Mix, Roche Diagnostics). The mRNA levels were normalized by glyceraldehyde‐3‐phosphate dehydrogenase (GAPDH) and the miRNA level was normalized by U6. Data were quantified by the 2^−ΔΔCt^ method. The primer sequences for target genes in this study are listed in Table 1.

**TABLE 1:** Primer information

Name of primer	Sequences
LncMEG3‐F‐homo	GAGAAAATGCAGGCCGAGAG
LncMEG3‐R‐homo	CCCCATTACTGTCCCCAAGT
LncMEG3‐F‐mus	GTGGACAATGGTGTCCAGGC
LncMEG3‐R‐mus	TTAACTCAGAGCGGGTCTCC
miR‐542‐3p‐F‐homo	UGUGACAGAUUGAUAACUGAAA
miR‐542‐3p‐R‐homo	GTGCAGGGTCCGAGGT
miR‐542‐3p‐F‐mus	TGTGACAGATTGATAACTGAAA
miR‐542‐3p‐R‐mus	GTGCAGGGTCCGAGGT
U6‐F‐homo	CTCGCTTCGGCAGCACA
U6‐R‐homo	AACGCTTCACGAATTTGCGT
U6‐F‐mus	CTCGCTTCGGCAGCACA
U6‐R‐mus	AACGCTTCACGAATTTGCGT
*SFRP1*‐F‐homo	AAAGCAAGGGCCATTTAGATTAG
*SFRP1*‐R‐homo	TTCTGGGCTTGACCTTAATTGTA
*Sfrp1*‐F‐mus	AAGCGAGTTTGCACTGAGGA
*Sfrp1*‐R‐mus	TACTGGCTCTTCACCTTGCG
*GAPDH*‐F‐homo	GACCTGCCGTCTAGAAAAACCTGC
*GAPDH*‐R‐homo	TCGCTGTTGAAGTCAGAGGAGACC
*Gapdh*‐F‐mus	GTCAACGGATTTGGTCTGTATT
*Gapdh*‐R‐mus	AGTCTTCTGGGTGGCAGTGAT
*p53*‐F‐homo	AAGTCTAGAGCCACCGTCCA
*p53*‐R‐homo	CTGGCATTCTGGGAGCTTCA
*p53*‐F‐mus	CCAAACTGCTAGCTCCCATCA
*p53*‐R‐mus	ATTTCATTGTAGGTGCCAGGGT

Abbreviation: F, forward primer; homo, human gene; mus, mouse gene; R, reverse primer.

### 2.4 Western blot assay

Cells were lysed with radio‐immunoprecipitation assay lysis buffer (Beyotime). The protein concentration was assayed by a bicinchoninic acid (BCA) kit (Beyotime). Next, the corresponding volume of proteins were appended with the loading buffer (Beyotime) and heated to denature the proteins. Afterwards, the proteins were separated by sodium dodecyl sulphate polyacrylamide gel electrophoresis, and the gel was electro‐transferred onto a polyvinylidene fluoride (PVDF) membrane, followed by 1–2 min of rinsing with the washing solution, 60‐min of blocking with the blocking solution at room temperature, or overnight at 4°C. After that, the membrane was subjected to 1 h‐incubation with primary antibodies against GAPDH (5174 S, 1:1000), SFRP1 (3534 S, 1:1000), E‐cadherin (ab231303, 1:1000, Abcam), N‐cadherin (14215 S, 1:1000), Vimentin (5741 S, 1:1000), Snail (3879 S, 1:1000), GSK‐3β (12456 S, 1:1000), p‐GSK‐3β (5558 S, 1:1000),β‐catenin (8480 S, 1:1000), and p53 (30313 S, 1:1000) (all from Cell Signaling Technology, Boston, MA, USA except for E‐cadherin) at room temperature. Next, the membrane was incubated for 1 h with secondary antibody at room temperature. Lastly, the membrane was dripped with developer solution and detected by Chemiluminescence imaging system (Bio‐Rad).

### 2.5 The cancer genome atlas (TCGA) analysis

Differentially expressed genes in GBM were analyzed by TCGA combined with Genotype‐Tissue Expression (GTEx). The transcriptome data of GBM were collected from the TCGA database (https://xenabrowser.net/datapages/), and the transcriptome data of normal tissues were collected from TCGA and GTEx. The limma package was used to compare and analyze the gene expression data of the GBM tissue samples and normal tissue samples obtained in the expression matrix. The screening threshold was set at |logFC| > 2 and adjusted *p* value <0.05. A volcano plot was created in the SangerBox website (http: https://sangerbox.com/Tool) to display the differentially expressed genes.

### 2.6 Actinomycin D test

Cells were seeded in 6‐well plates and cultured overnight with a low level of serum. Total RNA was extracted from the cells after they were treated with 2 g/L actinomycin D for 0, 12, and 24 h, followed by qRT‐PCR.

### 2.7 Cell counting kit‐8 (CCK‐8) assay

First, cells were treated with different concentrations of MTE (25, 50, 100, 200, and 400 mg/mL) for 48 h, and CCK‐8 was used to detect the effects of different concentrations of MTE on cell viability. Next, cells were treated with 200 mg/mL MTE for 0, 24, 48, and 72 h, and CCK‐8 was used to detect the effects of different treatment times on cell viability.

The cells treated with 200 mg/mL MTE for 48 h or the MTE‐treated (48 h) cells upon 48‐h transfection were inoculated onto a 96‐well plate (3 wells per group), and each well was seeded with the diluted cell suspension (1 × 10^5^ cells/mL, 100 μL). CCK‐8 reagent (10 μL, Dojindo) was supplemented to each well after the cells were incubated for 0, 24, 48, 72, and 96 h, respectively. Lastly, with another 2 h‐incubation, and the absorbance value of cells was measured at 450 nm.

### 2.8 Colony formation assay

Cells were harvested, trypsinized, centrifuged for 5 min (25°C, 1500 rpm), and added with the complete medium for resuspension. Each well was seeded with 500 cells in a 6‐well plate containing complete culture medium (2 mL), and the cells were subsequently cultured for 2–3 weeks. The culture was terminated when the cell colonies in the 6‐well plate were visible to the naked eye. Next, the culture medium was aspirated and the cells were subjected to two times of rinsing with phosphate‐buffered saline (PBS). Then, each well was appended with methanol (1.5 mL) and fixed for 15 min. After removing the methanol, the cells were slowly added with 1 mL of Giemsa staining solution along the well wall, and dyed in the dark for 20 min. Lastly, the Giemsa staining solution was washed away with running water, and the 6‐well plate was dried by placing upside down on clean absorbent paper, followed by counting the number of colonies.

### 2.9 Wound healing assay

Cells (2 × 10^6^ cells/well) were seeded onto 6‐well plates, 3 wells in each group, and the cells were then maintained for 24 h until the cell monolayer was confluent. Next, the monolayer cells were scratched with a sterile pipette tip (200 μL), with the floating cells washed away with PBS. The cells were then cultured with serum‐free medium for another 24 h. The scratch distance was observed under the microscope and photographed at 0 h and the 24th h after culture. The cell migration was analyzed according to the change of scratch distance after 0‐ and 24‐h culture, namely, (0‐h scratch distance ‐ 24‐h scratch distance)/0‐h scratch distance.

### 2.10 Transwell invasion assay

Transwell plates (Corning Costar) were precoated overnight at 37°C with 0.1 mL 200 μg/mL Matrigel (BD Bioscience, Franklin Lakes, NJ, USA). Then, cells in the logarithmic growth phase were made into single‐cell suspension and evenly seeded into 6‐well plates (3 wells per group) for incubation at 37°C in 5% CO_2_ until the cell confluence reached 70%–90%. Next, the cells were treated according to their groupings and cultured for another 24 h. The cells were trypsinized, washed twice with PBS, and resuspended in serum‐free DMEM. The Transwell basolateral chamber was supplemented with 600 μL of 10% FBS‐containing DMEM, and the upper chamber was added with 100 μL of prepared cell suspension, followed by 24‐h cell incubation. The Transwell insert was then taken out, and the supernatant was discarded. The cells remaining on the upper side of the Transwell membrane were removed with a cotton swab. The invasive cells were fixed with 4% paraformaldehyde and dyed with 0.5% crystal violet solution, both for 10 min. The number of invasive cells was captured using a light microscope. Cells in five randomly selected areas were counted in each group. All measurements were independently repeated thrice.

### 2.11 Dual‐luciferase reporter gene assay

The binding site of miR‐542‐3p to SFRP1, together with miR‐542‐3p to lncMEG3 was predicted by the online websites starBase (http://starbase.sysu.edu.cn/) and miRDB (http://www.mirdb.org/). According to the predicted results, the wild type and mutant type sequences (WT‐SFRP1 and Mut‐SFRP1/WT‐MEG3 and Mut‐MEG3) of the binding site were designed and synthesized, respectively. Subsequently, The WT and Mut sequences of the binding site were inserted into a luciferase reporter gene vector (pGL3‐Promoter), and then cotransfected into HEK293T cells (Shanghai Sixin Biotechnology Co., Ltd.) with miR‐542‐3p Agomir (30 nM) or Agomir NC (30 nM), respectively. Firefly luciferase activity was standardized with the Renilla luciferase activity. The ratio of Firefly luciferase activity to Renilla luciferase activity was considered as the relative activity of luciferase.

### 2.12 Fluorescent in‐situ hybridization (FISH) assay

FISH experiments were conducted for detecting the colocalization of lncMEG3 and miR‐542‐3p or miR‐542‐3p and SFRP1 in human U87 and U251 glioma cells and NHAs. Fluorescein isothiocyanate (FITC)‐labeled lncMEG3 and SFRP1 and cy3‐labeled miR‐542‐3p probes were purchased from Biosense (Guangzhou, China). Analysis was evaluated using a FISH kit. Nuclei were stained with 4′‐6‐diamidino‐2‐phenylindole for 20 min, and the images were finally analyzed by a fluorescence microscopy.

### 2.13 RNA immunoprecipitation (RIP)

Sample preparation: cells were harvested, washed twice with precooled PBS, and centrifuged for 5 min, followed by adding an equal volume of RIP lysis buffer.

Magnetic bead preparation: the magnetic beads were resuspended, and 50 μL of the magnetic bead suspension was appended to each centrifuge tube, followed by adding with 500 μL RIP Wash Buffer. After the removal of the supernatant, the magnetic beads were resuspended with 100 μL RIP Wash Buffer, and incubated for 30 min with about 5 μg Ago2 antibody (2897 S, 1:50, Cell Signaling Technology, Boston, USA). After the removal of the supernatant, the magnetic beads were added and washed with 500 μL RIP Wash Buffer twice. The prepared magnetic bead‐antibody complexes were added with 900 μL RIP Immunoprecipitation Buffer to each tube. Afterward, the prepared cell lysate was quickly thawed, and then centrifuged for 10 min (14,000 rpm, 4°C). The supernatant (100 μL) was supplemented into the magnetic bead‐antibody complex to make a total volume of 1 mL, followed by centrifugation to discard the supernatant. Subsequently, the magnetic bead‐antibody‐RNA complex was added with 500 μL RIP Wash Buffer, and placed on the magnetic stand after vibration for the removal of the supernatant.

RNA purification: Each sample was appended with 150 μL Proteinase K Buffer to suspend the magnetic bead‐antibody complex, followed by incubation at 55°C for 30 min, which was then placed on a magnetic stand for the removal of the supernatant. After RNA extraction, lncMEG3, miR‐542‐3p, and SFRP1 expression was tested via the qRT‐PCR.

### 2.14 Experimental animals

BALB/c nude mice (*n* = 42, specific pathogen free (SPF)‐grade, 4–6 weeks old, 16 ± 2 g) were got from the Shanghai Laboratory Animal Center, Chinese Academy of Sciences (Shanghai, China). All padding, drinking water, pellet feed, and other items were autoclaved. These animals were reared in a sterile laminar flow room of SPF grade under (22–26°C) temperature and (55 ± 5%) humidity.

### 2.15 Tumor models

To verify the effect of MTE on gliomas in situ in nude mice, a glioma model was established with the commonly used human glioma cell line U251. U251 cells were infected with lentiviruses dual‐labeled with luciferase and green fluorescent protein (GFP), and the cells stably expressing GFP fluorescence were screen out by flow cytometry. Nude mice were assigned into 7 groups (*n* = 6) in a random fashion: control (CON), MTE, MTE + sh‐NC, MTE + sh‐MEG3, MTE + sh‐NC + Antagomir NC, MTE + sh‐MEG3 + Antagomir NC, and MTE + sh‐MEG3 + miR‐Antagomir groups. The CON and MTE groups were injected with U251‐GFP‐Luc cell suspension, the MTE + sh‐NC group was injected with NC shRNA‐transfected and GFP‐Luc‐labeled U251 cells, and the MTE + sh‐MEG3 group was injected with lncMEG3 shRNA‐transfected and GFP‐Luc‐labeled U251 cells. The MTE + sh‐NC + Antagomir NC group was injected with NC shRNA‐ and Antagomir NC‐transfected and GFP‐Luc‐labeled U251 cells, the MTE + sh‐MEG3 + Antagomir NC group was injected with lncMEG3 shRNA‐ and Antagomir NC‐transfected and GFP‐Luc‐labeled U251 cells, and the MTE + sh‐MEG3 + miR‐Antagomir group was injected with lncMEG3 shRNA‐ and miR‐542‐3p Antagomir‐transfected and GFP‐Luc‐labeled U251 cells.

The nude mice were anesthetized by pentobarbital sodium (60 mg/kg) via the intraperitoneal injection. After that the nude mice’ head skin was sterilized with iodophor, and these mice were then fixed in the center of the tripod with a stereotaxic instrument. Subsequently, the skin of the head was incised vertically 3 mm to the right of the midline of the head and exposing the skull at the junction of the line connecting the two ears. Next, according to the intersection of bilateral eyes and ears 2 mm to the right, the skull of nude mice was drilled by using a dental drill. The cell suspensions of U251‐GFP‐Luc (1 × 10^6^ cells) were absorbed by a sterilized microsampler and then fixed on the stereotaxic instrument.^22^, ^23^ The needle was inserted along the borehole (about 3 mm), and the stereotaxic instrument was started to slowly inject the cell suspension after receding 1 mm. Post the injection, the drill hole was closed with bone wax, followed by the suturing of skin incision, and the sterilization of wound surface. After the nude mice were recovered from anesthesia, they were put back into the cage to continue feeding. After the injection with cell suspension, the mice were intraperitoneally injected with drugs every 2 days. Specifically, the mice in the CON were injected with 200 μL of normal saline, and the MTE, MTE + sh‐NC, MTE + sh‐MEG3, MTE + sh‐NC + Antagomir NC, MTE + sh‐MEG3 + Antagomir NC, and MTE + sh‐MEG3 + miR‐Antagomir groups were injected with 200 μL of MTE (100 mg/kg). The nude mice were euthanatized after 28 days, and the tumor tissues in their brains were collected and stored at −80°C.

### 2.16 In vivo imaging system

The mice's late reaction was regularly observed. The mice were treated with luciferase substrate (15 mg/mL, 10 μL/g) via the intraperitoneal injected on the 0, 7th, 14th, 21st, and 28th day after U251‐GFP‐Luc cell transplantation. The luminescence signal changes were viewed by a small in vivo imager (IVIS Spectrum, Caliper, USA), and the tumor formation and growth state in the brain were assessed.

### 2.17 Immunohistochemistry

The tumor tissues were fixed for 48 h with 4% paraformaldehyde, followed by the preposition of paraffin sections (4 μm). In brief, the paraffin sections were heated for 30 min, dewaxed with conventional xylene, and incubated with Ki‐67 rabbit monoclonal antibody (ab16667, 1:200, Abcam) and the secondary antibody. Following washing three times in PBS, the sections were developed with diaminobenzidine, which was terminated 1–3 min later. Subsequently, the sections (nucleus) were stained with hematoxylin for 3 min, followed by dehydration, permeabilization, and blocking. Ki‐67 showing yellow or brown granules in the nucleus was regarded to be positive. The percentage of positive cells was counted in the selected 5 high‐power fields.

### 2.18 Statistical analysis

Data were processed with GraphPad prism8 software, and all data were reported as mean ± standard deviation. The normality of the data was analyzed using the Shapiro–Wilk test, and the homogeneity of variance was analyzed using the Bartlett's test. For data with normal distribution and homogeneity of variance, two‐group comparisons were performed using the *t*‐test, and multiple‐group comparisons were performed using the one‐way analysis of variance (ANOVA) and Tukey's post hoc test. *p* less than 0.05 indicates a significant difference.

## 3 RESULTS

### 3.1 MTE treatment suppresses glioma cell aggressive behaviors

**FIGURE 1:**
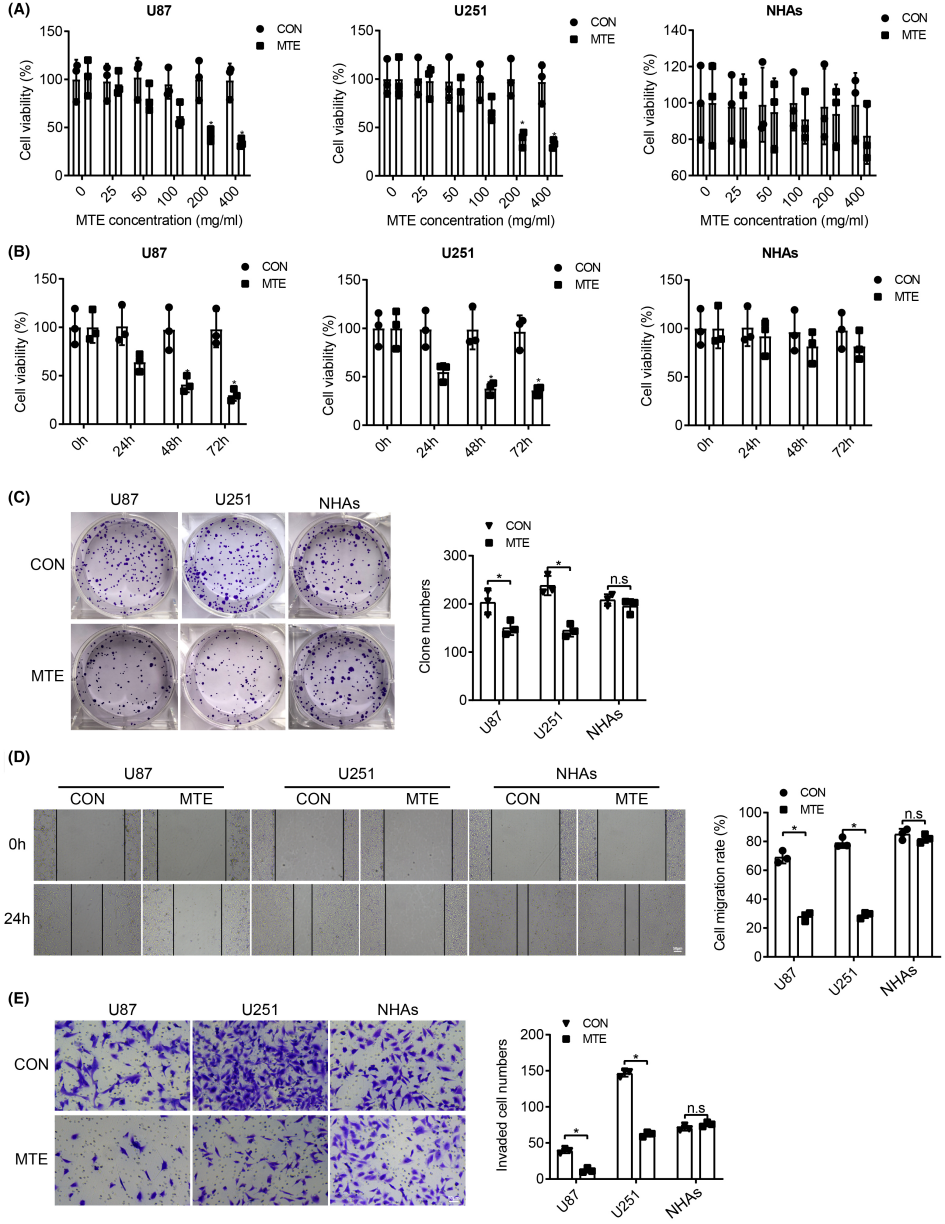
Proliferation, migration, and invasion abilities of glioma cells are suppressed by the treatment of MTE. A. CCK‐8 assay was performed to detect the viability of U87, U251, and NHAs pretreated with different concentrations of MTE for 48 h. B. CCK‐8 assay was carried out to detect the viability of U87, U251, and NHAs pretreated with 200 mg/mL MTE for 0, 24, 48, and 72 h. After pretreatment of U87, U251, and NHAs with 200 mg/mL MTE for 48 h: C. Cell proliferation was detected by colony formation assay. D. Cell migration was determined by wound healing assay. E. Cell invasion was tested by Transwell assay. n.s refers to no significance; *p < 0.05. *N* = 3.

First, glioma cells (U87 and U251) and NHAs were treated with different concentrations of MTE (25, 50, 100, 200, and 400 mg/mL) for 48 h. The cell proliferation activity was measured by CCK‐8 assay and the results elucidated that MTE with a concentration of or more than 200 mg/mL would reduce the proliferation activity of U87 and U251 to 50% below but not affect that of NHAs (Figure 1A). Next, U87, U251, and NHAs were treated with 200 mg/mL MTE for different times (0, 24, 48, and 72 h). The treatments with 200 mg/mL MTE for 48 and 72 h significantly inhibited the proliferative capability of U87 and U251 but did not affect that of NHAs (Figure 1B). Therefore, 200 mg/mL MTE was selected to treat U87, U251, and NHAs for 48 h in the subsequent experiments. Colony formation, wound healing, and Transwell invasion assays were conducted to test the proliferation, migration, and invasion of the treated or nontreated cells. MTE treatment weakened the proliferation (Figure 1C), migration ability (Figure 1D), and invasion ability (Figure 1E) of glioma cells, while not affecting NHAs. These results uncover that MTE can effectively hinder the aggressive behaviors of glioma cells but have no obvious proliferation inhibition and toxicity to NHAs.

### 3.2 MTE treatment impedes proliferative, migratory, and invasive properties in glioma cells through upregulating lncMEG3 expression

**FIGURE 2:**
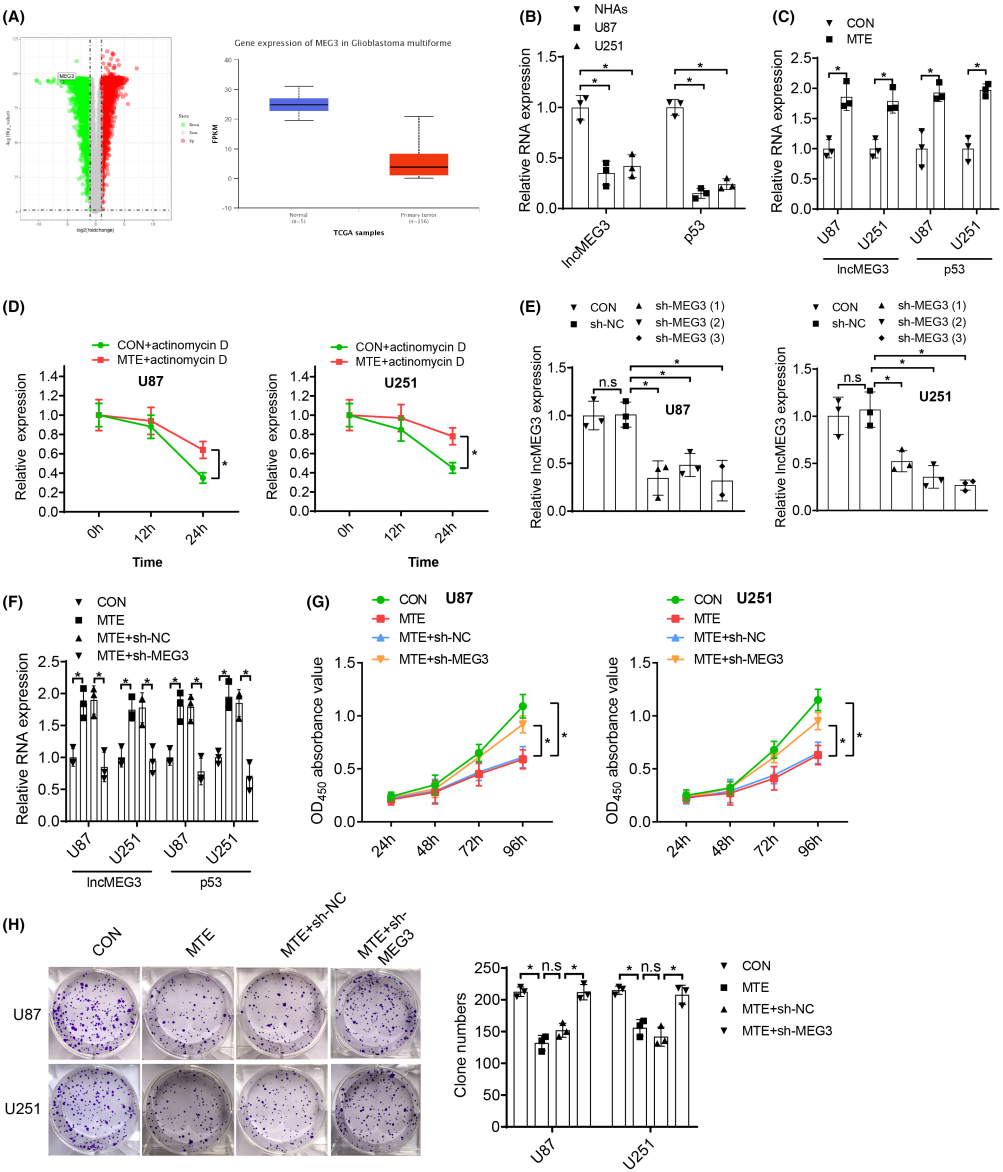
MTE treatment impedes proliferation of glioma cells through upregulating lncMEG3 expression. A. TCGA data and UALCAN analysis of lncMEG3 expression in GBM. B. qRT‐PCR was performed to test lncMEG3 and p53 expression in U87 and U251 glioma cells and NHAs. After pretreatment of U87 and U251 cells with 200 mg/mL MTE for 48 h: C. LncMEG3 and p53 expression was detected by qRT‐PCR. D. The stability of lncMEG3 was analyzed. E. qRT‐PCR for the detection of lncMEG3 and p53 expression in U87 and U251 cells transfected with different shRNAs in the lncMEG3 shRNA screening experiment. U87 and U251 cells were pretreated with 200 mg/mL MTE for 48 h, followed by transfection with lncMEG3 shRNA: F. LncMEG3 and p53 expression was determined by qRT‐PCR. G. CCK‐8 assay was implemented to detect the cell viability of U87 and U251 cells. H. Cell proliferation of U87 and U251 cells was detected by colony formation assay. n.s refers to no significance; *p < 0.05. *N* = 3.

Loss of lncMEG3 expression is witnessed in many types of tumors, and many functions of lncMEG3 in glioma have been defined. As a tumor suppressor, lncMEG3 mainly regulates cell adhesion, epithelial‐mesenchymal transition (EMT), and cell proliferation.^24^ The tumor suppressor p53 plays a central role in tumor suppression, which mediates the functions of many other tumor suppressors. It has been reported that lncMEG3 mediates the upregulation of p53 expression to inhibit tumor growth.^25^ In addition, we used UALCAN (http://ualcan.path.uab.edu/analysis.html) to access data from TCGA, and the analysis showed that lncMEG3 was poorly expressed in GBM (Figure 2A). Therefore, we speculated that MTE may affect lncMEG3 and p53 expression in glioma cells to exert a tumor‐suppressive effect.

First, lncMEG3 and p53 expression was tested in glioma cells and NHAs, which deciphered lower expression of lncMEG3 and p53 in glioma cells versus NHAs (Figure 2B). Next, lncMEG3 and p53 expression in glioma cells with/without MTE was determined by qRT‐PCR, with the outcomes highlighted that MTE treatment increased lncMEG3 and p53 expression in U87 and U251 cells (Figure 2C). To further evaluate the effect of MTE on the stability of lncMEG3, we pretreated U87 and U251 cells with 200 mg/mL MTE for 48 h and then treated them with actinomycin D for different times. The qRT‐PCR analysis showed that MTE pretreatment prolonged the half‐life of lncMEG3 in U87 and U251 cells (Figure 2D).

**FIGURE 3:**
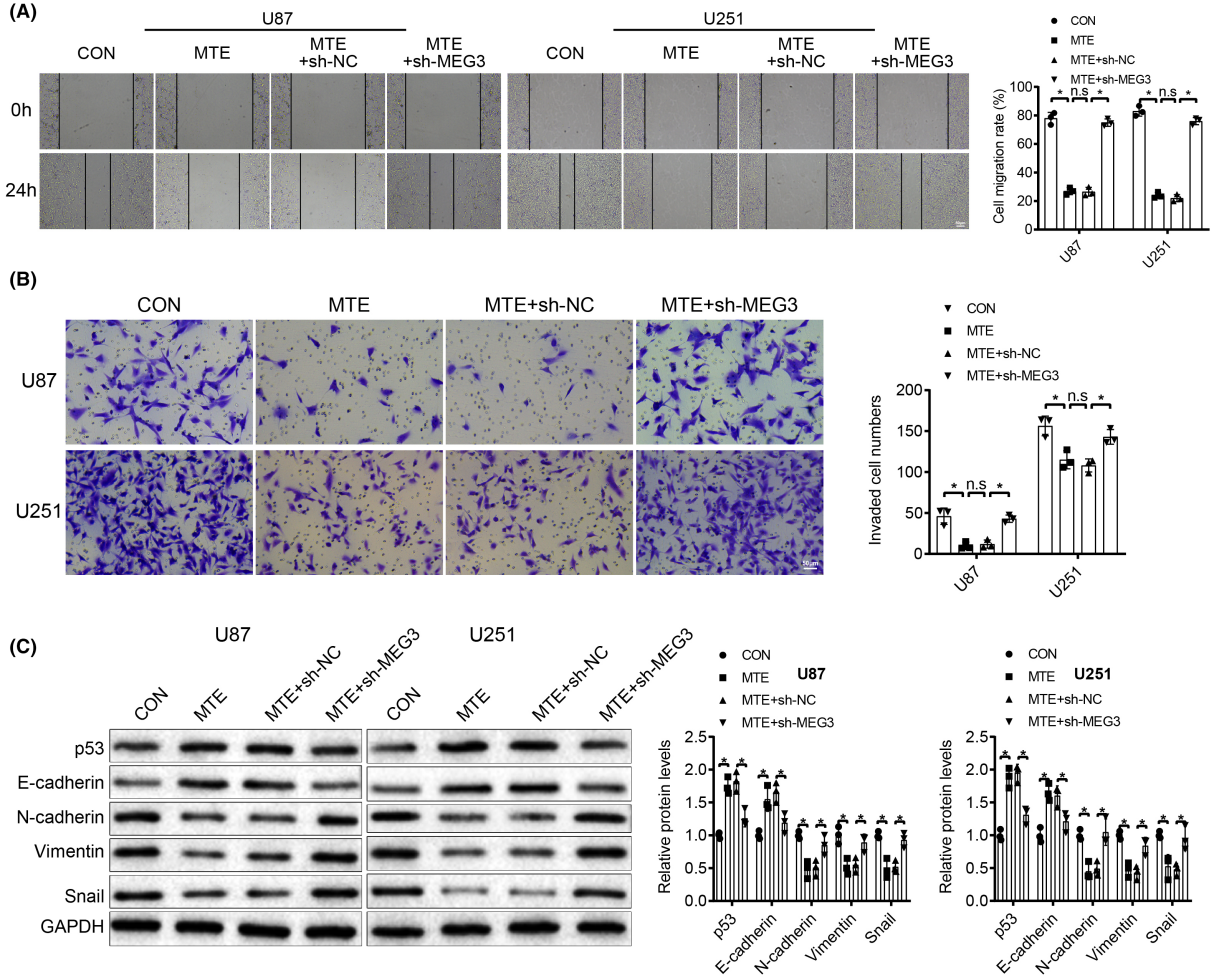
MTE treatment inhibits migration and invasion of glioma cells through upregulating lncMEG3 expression. U87 and U251 cells were pretreated with 200 mg/mL MTE for 48 h, followed by transfection with lncMEG3 shRNA: A. Cell migration of U87 and U251 cells was determined by wound healing assay. B. Cell invasion of U87 and U251 cells was tested by Transwell assay. C. Expression levels of p53 protein and EMT‐related proteins E‐cadherin, N‐cadherin, Vimentin, and Snail in glioma cells were measured by western blot analysis. n.s refers to no significance; *p < 0.05. *N* = 3.

Next, U87 and U251 cells we pretreated with MTE and then transfected with lncMEG3 shRNA or NC shRNA. In the screening experiment of lncMEG3 shRNAs, sh‐MEG3^3^ had the best interference effect, which was used for subsequent experiments (Figure 2E). The malignant phenotypes of glioma cells upon lncMEG3 shRNA treatment were detected by CCK‐8, colony formation, wound healing, and Transwell invasion assays. The results indicated that MTE treatment elevated lncMEG3 and p53 expression, which could be neutralized with the further transfection of sh‐MEG3 (Figure 2F). Additionally, MTE treatment restricted the proliferation (Figure 2G,H), migration (Figure 3A) and invasion (Figure 3B) of U87 and U251 cells; the restriction could be offset by the transfection of sh‐MEG3 (Figures 2G,H and 3A,B). Furthermore, p53 protein and EMT‐related proteins E‐cadherin, N‐cadherin, Vimentin, and Snail in glioma cells were tested, and the corresponding results revealed that MTE treatment resulted in increased expression levels of E‐cadherin and p53 and decreased expression levels of N‐cadherin, Vimentin, and Snail, and that further transfection of sh‐MEG3 could offset the MTE treatment‐mediated effects on the expression levels of these proteins (Figure 3C). It is suggested that MTE treatment suppresses malignant behaviors of glioma cells via increasing lncMEG3 and p53 expression.

### 3.3 LncMEG3 and SFRP1 competitively bind with miR‐542‐3p

**FIGURE 4:**
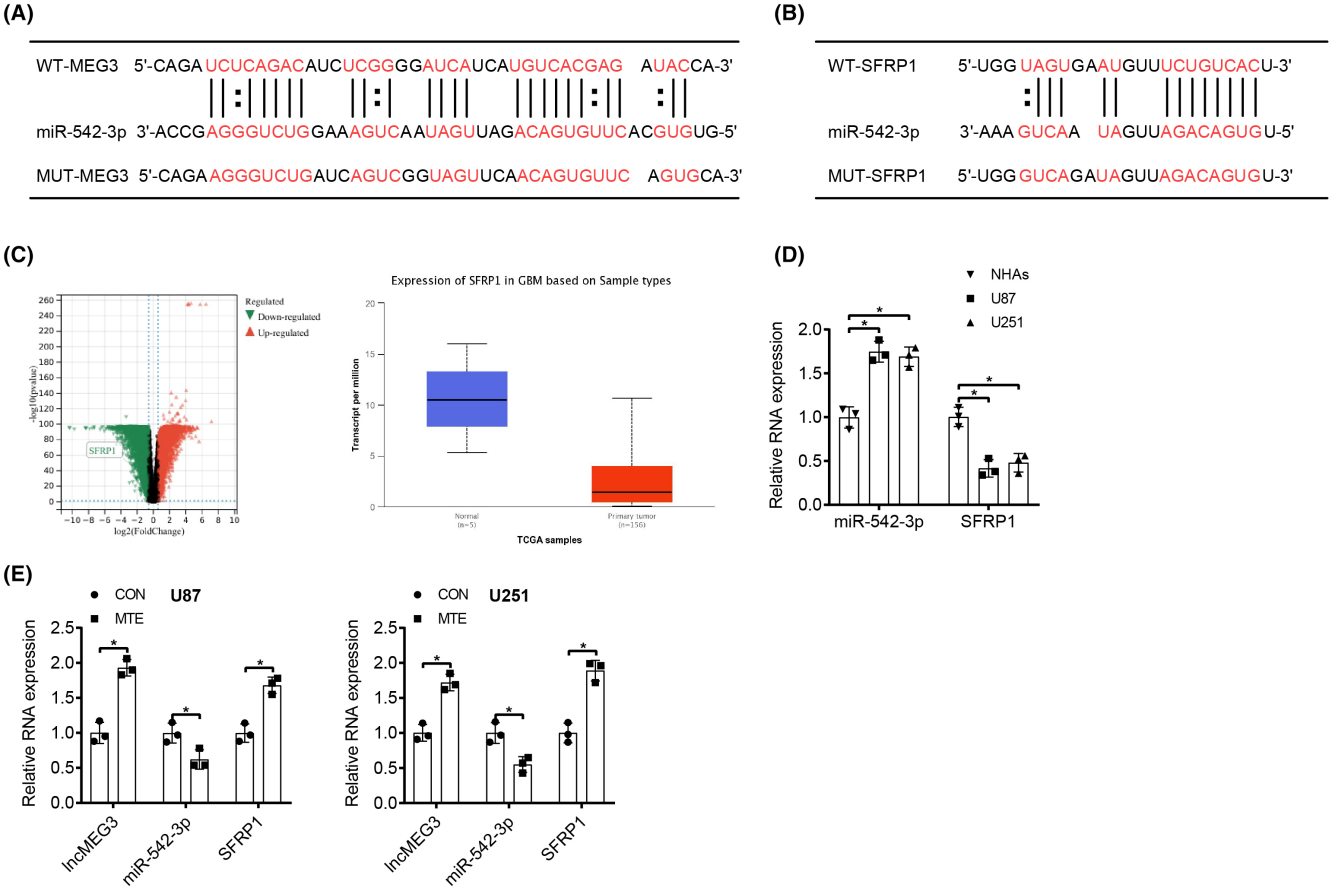
LncMEG3 and SFRP1 competitively bind to miR‐542‐3p. A. The binding of lncMEG3 and miR‐542‐3p was predicted by Starbase. B. The binding of miR‐542‐3p and SFRP1 was predicted by miRDB. C. UALCAN analysis of TCGA data of SFRP1 expression in GBM. D. Expression levels of miR‐542‐3p and SFRP1 in NHAs and U87 and U251 glioma cells were tested by qRT‐PCR. E. Expression levels of lncMEG3, miR‐542‐3p, and SFRP1 in MTE‐treated U87 and U251 cells were determined by qRT‐PCR. *p < 0.05. *N* = 3.

**FIGURE 5:**
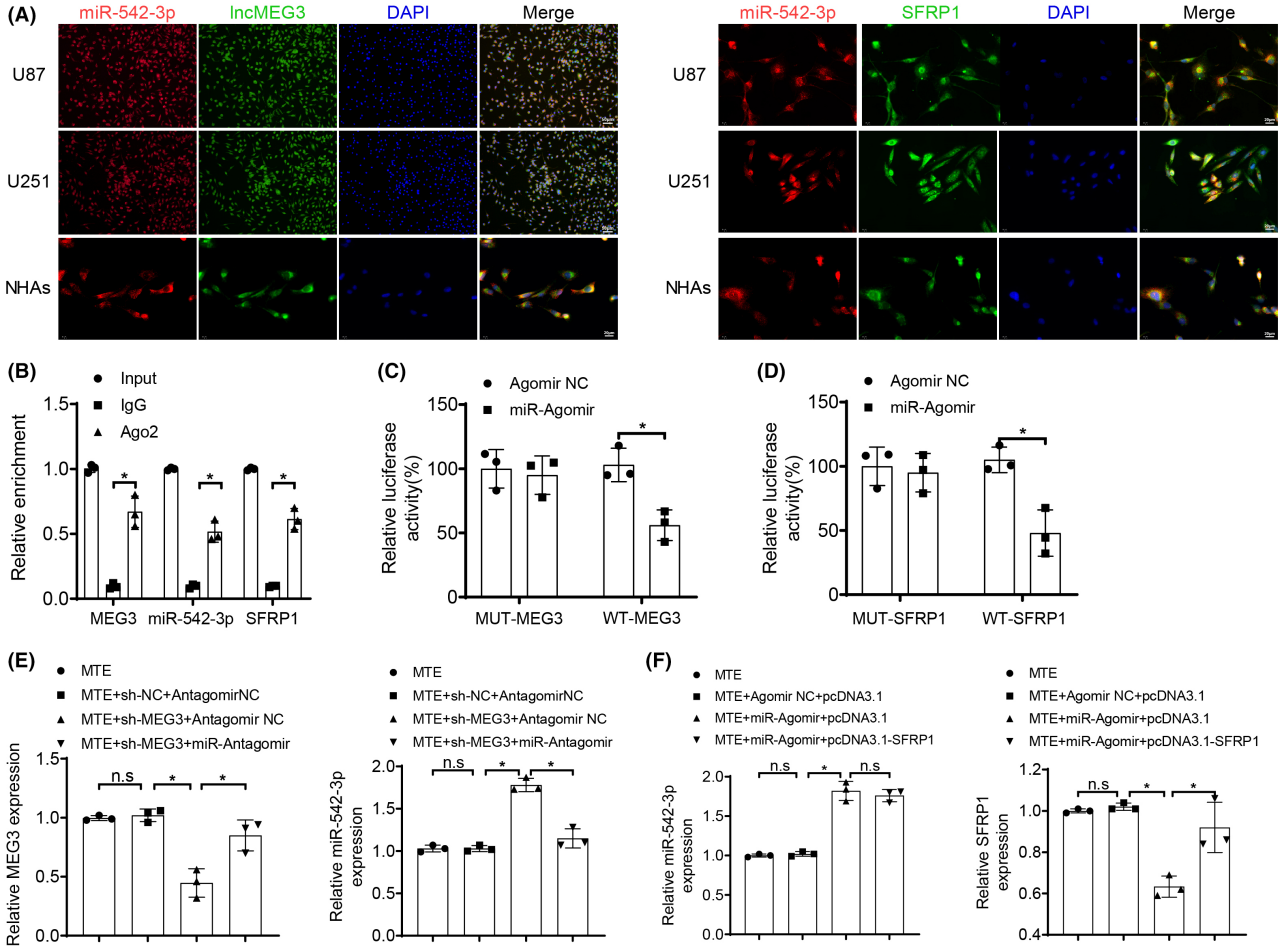
LncMEG3 and SFRP1 competitively bind to miR‐542‐3p. A. The localization of lncMEG3, miR‐542‐3p, and SFRP1 was detected by FISH assay. B. The binding of miR‐542‐3p to lncMEG3 and SFRP1 was examined by RIP assay. C‐D. Dual‐luciferase reporter gene assay was carried out to further verify the relationships among lncMEG3, miR‐542‐3p, and SFRP1. On the basis of MTE treatment, U87 cells were continued to be treated with lncMEG3 shRNA alone or cotransfected with miR‐542‐3p Antagomir: E. LncMEG3 and miR‐542‐3p expression was tested by qRT‐PCR. On the basis of MTE treatment, U87 cells were further transfected with miR‐542‐3p Agomir alone or cotransfected with pcDNA3.1‐SFRP1: F. miR‐542‐3p and SFRP1 expression was tested by qRT‐PCR. n.s refers to no significance; *p < 0.05. *N* = 3.

Starbase website (http://starbase.sysu.edu.cn/) was searched and there were binding sites between miR‐542‐3p and lncMEG3 (Figure 4A). Meanwhile, a binding site between miR‐542‐3p and SFRP1 was searched by using miRDB website (http://www.mirdb.org/) (Figure 4B), and the UALCAN analysis of TCGA database also demonstrated that SFRP1 was lowly expressed in GBM (Figure 4C). Based on this, we could speculate that the MTE treatment suppressed the malignant behaviors of glioma cells through upregulating lncMEG3, reducing miR‐542‐3p expression, and finally upregulating SFRP1 expression. To confirm this conjecture, we first performed qRT‐PCR for the detection of miR‐542‐3p and SFRP1 expression in NHAs and U87 and U251 glioma cells, and a high expression of miR‐542‐3p and a low expression of SFRP1 were observed in U87 and U251 cells in comparison to NHAs (Figure 4D). Next, the expression levels of lncMEG3, miR‐542‐3p and SFRP1 in U87 and U251 cells with the addition of MTE were determined, and the results disclosed that the MTE treatment led to an increased expression of lncMEG3 and SFRP1 and a decreased expression of miR‐542‐3p (Figure 4E). According to the FISH results, we found that miR‐542‐3p colocalized with lncMEG3 and SFRP1 in the cytoplasm in U87, U251, and NHAs (Figure 5A). RIP assay was then performed, which suggested that a large amount of lncMEG3, miR‐542‐3p and SFRP1 were witnessed in cells with the addition of Ago2 antibody, while with the addition of IgG, lncMEG3, miR‐542‐3p and SFRP1 were barely detected (Figure 5B). Subsequently, a dual‐luciferase reporter gene assay was examined to further affirm the relationships among lncMEG3, miR‐542‐3p and SFRP1. The results revealed that cotransfection of miR‐Agomir and WT‐MEG3 or WT‐SFRP1 vectors markedly attenuated the luciferase activity. In contrast, cotransfection of miR‐Agomir and MUT‐MEG3 or MUT‐SFRP1 vectors demonstrated no effect on luciferase activity (Figure 5C,D).

On account of MTE treatment, U87 cells were continued to receive treatment of lncMEG3 shRNA alone or cotransfected with miR‐542‐3p Antagomir to further decipher the binding relationship of lncMEG3 and miR‐542‐3p. The observations unveiled that no change was witnessed in lncMEG3 and miR‐542‐3p between the MTE + sh‐NC + Antagomir NC group and the MTE group; lncMEG3 expression was diminished and miR‐542‐3p expression was enhanced in the MTE + sh‐MEG3 + Antagomir NC group versus the MTE + sh‐NC + Antagomir NC group; elevated lncMEG3 expression and reduced miR‐542‐3p expression were witnessed in the MTE + sh‐MEG3 + miR‐Antagomir group versus the MTE + sh‐MEG3 + Antagomir NC group (Figure 5E). The aforesaid results further indicate that lncMEG3 could combine with miR‐542‐3p, and the MTE negatively modulated miR‐542‐3p expression by upregulating lncMEG3. Lastly, on the basis of MTE treatment, U87 cells were further transfected with miR‐542‐3p Agomir alone or cotransfected with pcDNA3.1‐SFRP1 (grouped as: MTE, MTE + Agomir NC + pcDNA3.1, MTE + miR‐Agomir + pcDNA3.1, and MTE + miR‐Agomir + pcDNA3.1‐SFRP1 groups), with miR‐542‐3p and SFRP1 expression in U87 cells being detected by qRT‐PCR, which exerted no change between the MTE + Agomir NC + pcDNA3.1 group and the MTE group; a high miR‐542‐3p expression and a low SFRP1 expression were found in the MTE + miR‐Agomir + pcDNA3.1 group in comparison to the MTE + Agomir NC + pcDNA3.1 group; however, compared with the MTE + miR‐Agomir + pcDNA3.1 group, SFRP1 expression was increased in the MTE + miR‐Agomir + pcDNA3.1‐SFRP1 group, with no change in miR‐542‐3p expression (Figure 5F). It validated that SFRP1 was a target gene of miR‐542‐3p and miR‐542‐3p negatively modulated SFRP1 expression. All these results imply that lncMEG3 and SFRP1 can competitively bind to miR‐542‐3p, and lncMEG3 upregulates SFRP1 expression by enhancing miR‐542‐3p expression.

### 3.4 Overexpression of SFRP1 blocks the activation of the Wnt/β‐catenin pathway, thereby suppressing the malignant phenotype of glioma cells

**FIGURE 6:**
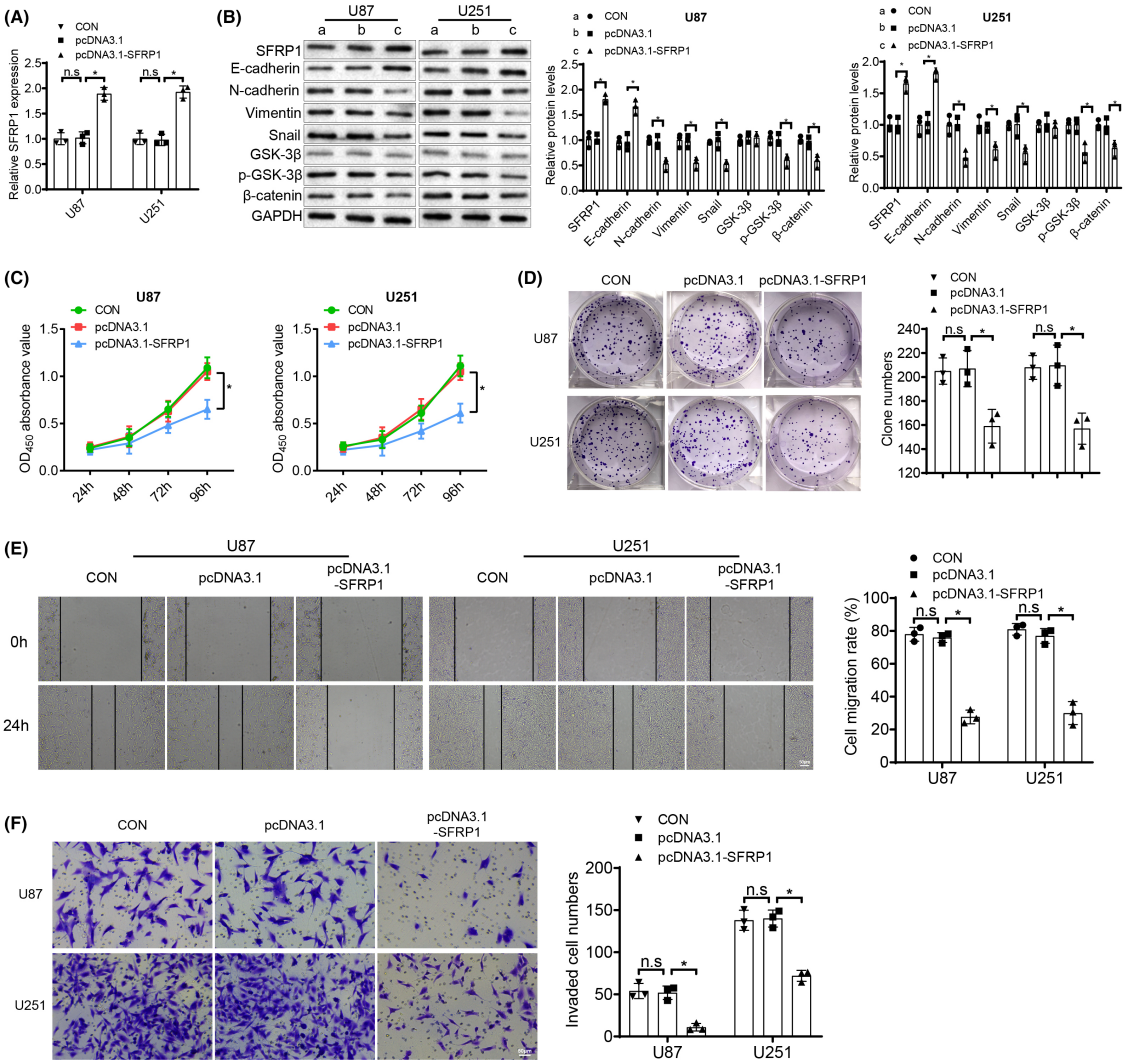
Overexpression of SFRP1 inhibits the Wnt/β‐catenin pathway activation and further represses glioma cell proliferation, migration, invasion and EMT. In response to pcDNA3.1‐SFRP1 or pcDNA3.1 treatment in U87 and U251 cells: A. SFRP1 expression was tested by qRT‐PCR. B. The Wnt/β‐catenin pathway‐associated factors (GSK‐3β, p‐GSK‐3β and β‐catenin) and EMT‐related factors (E‐cadherin, N‐cadherin, Vimentin and Snail) were detected by western blot analysis. C. Cell viability was measured by CCK‐8 assay. D. Cell proliferation ability was detected by colony formation assay. E. Cell migration ability was determined by wound healing assay. F. Cell invasion ability was tested by Transwell assay. n.s refers to no significance; *p < 0.05. *N* = 3.

Emerging evidence has shown that SFRP1 can inhibit the Wnt/β‐catenin pathway and further impact cell proliferation and other abilities.^26^, ^27^ Therefore, we also verified the association of this pathway with SFRP1 expression in U87 and U251 glioma cells. U87 and U251 glioma cells upon pcDNA3.1‐SFRP1 treatment was found to have elevated SFRP1 and E‐cadherin expression and reduced p‐GSK‐3β, β‐catenin, N‐cadherin, Vimentin, and Snail expression, with no change in GSK‐3β expression (Figure 6A,B). In response to pcDNA3.1‐SFRP1 treatment, the capabilities of proliferation (Figure 6C,D), migration (Figure 6E) and invasion (Figure 6F) were revealed to be diminished. To conclude, restoration of SFRP1 can restrict the malignant phenotype of glioma cells by restraining the activation of the Wnt/β‐catenin pathway.

### 3.5 MTE impedes glioma cell malignant behaviors by blocking the Wnt/β‐catenin pathway via miR‐542‐3p‐SFRP1 feedback loop

**FIGURE 7:**
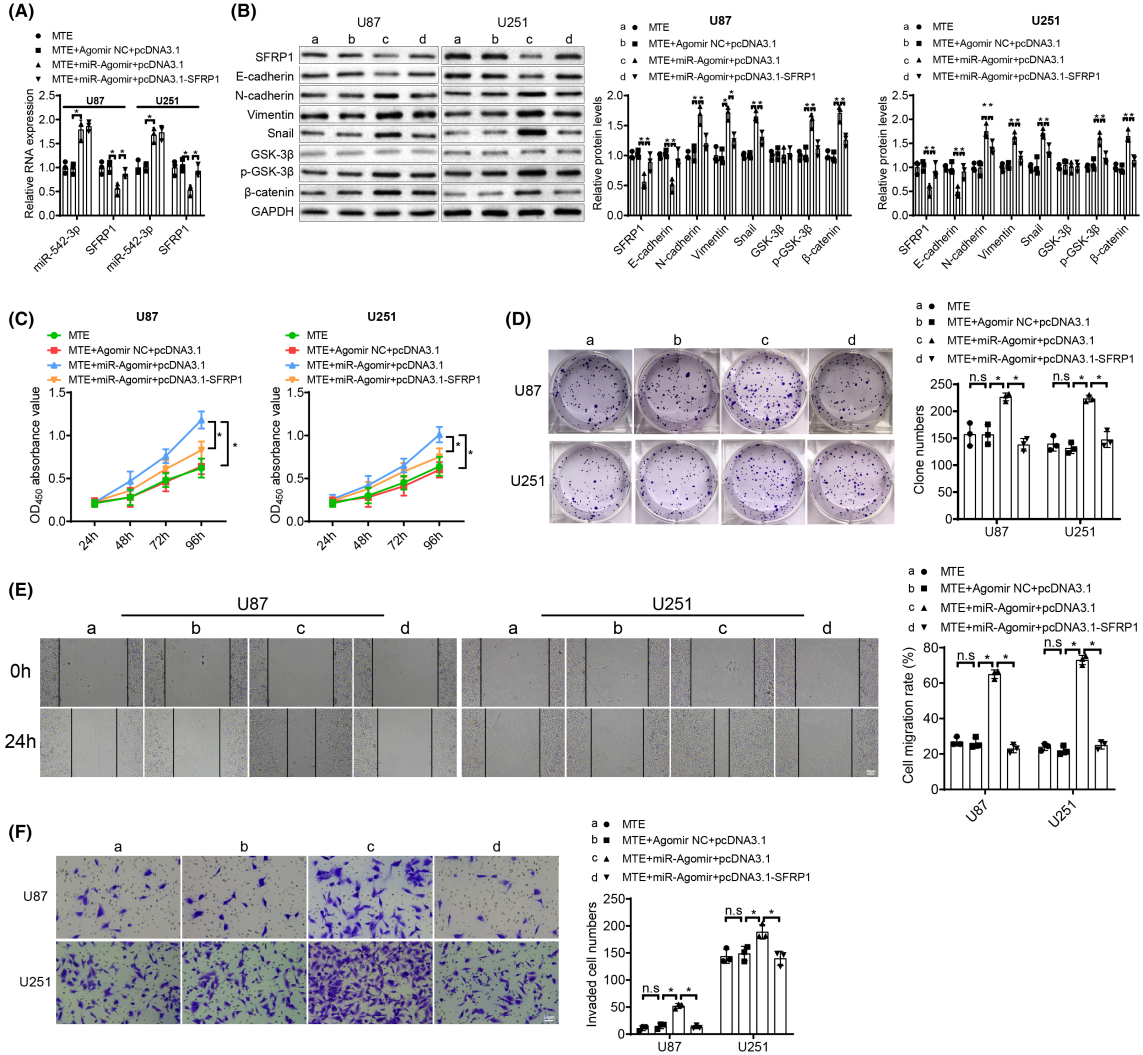
MTE suppresses the malignant behaviors of glioma cells by decreasing miR‐542‐3p, enhancing SFRP1 and blocking the Wnt/β‐catenin pathway. U87 and U251 cells with MTE treatment were then transfected with miR‐542‐3p Agomir and pcDNA3.1‐SFRP1: A. miR‐542‐3p and SFRP1 expression was tested by qRT‐PCR. B. The Wnt/β‐catenin pathway‐associated factors (GSK‐3β, p‐GSK‐3β and β‐catenin) and EMT‐related factors (E‐cadherin, N‐cadherin, Vimentin and Snail) were determined by western blot analysis. C. Cell viability was measured by CCK‐8 assay. D. Cell proliferation ability was detected by colony formation assay. E. Cell migration ability was determined by wound healing assay. F. Cell invasion ability was tested by Transwell assay. n.s refers to no significance; *p < 0.05. *N* = 3.

We next verified whether the MTE‐mediated inhibition of glioma cells was acted by blocking the Wnt/β‐catenin pathway, decreasing miR‐542‐3p expression and elevating SFRP1 expression. U87 and U251 cells with MTE treatment were then transfected with miR‐542‐3p Agomir and pcDNA3.1‐SFRP1. It was observed that reduced SFRP1 and E‐cadherin expression while elevated miR‐542‐3p, p‐GSK‐3β, β‐catenin, N‐cadherin, Vimentin, and Snail expression were witnessed in the MTE + miR‐Agomir + pcDNA3.1 group in comparison to the MTE + Agomir NC + pcDNA3.1 group; there were higher expression of SFRP1 and E‐cadherin while lower expression of p‐GSK‐3β, β‐catenin, N‐cadherin, Vimentin and Snail expression in the MTE + miR‐Agomir + pcDNA3.1‐SFRP1 group versus the MTE + miR‐Agomir + pcDNA3.1 group, with no change in miR‐542‐3p expression (Figure 7A,B). Afterward, the malignant behaviors of U87 and U251 cells were analyzed, and the obtained findings demonstrated that the cell proliferative activity (Figure 7C,D), migration activity (Figure 7E), and invasion ability (Figure 7F) were promoted in the MTE + miR‐Agomir + pcDNA3.1 group in comparison to the MTE + Agomir NC + pcDNA3.1 group; there were diminished cell proliferative activity (Figure 7C,D), migration activity (Figure 7E), and invasion ability (Figure 7F) in the MTE + miR‐Agomir + pcDNA3.1‐SFRP1 group versus the MTE + miR‐Agomir + pcDNA3.1 group. To sum up, MTE impedes the malignant behaviors of glioma cells by suppressing the Wnt/β‐catenin pathway via the miR‐542‐3p‐SFRP1 feedback loop.

### 3.6 MTE upregulates SFRP1 and blocks the Wnt/β‐catenin pathway activation via the lncMEG3/miR‐542‐3p axis, thereby restricting glioma cell malignant behaviors

**FIGURE 8:**
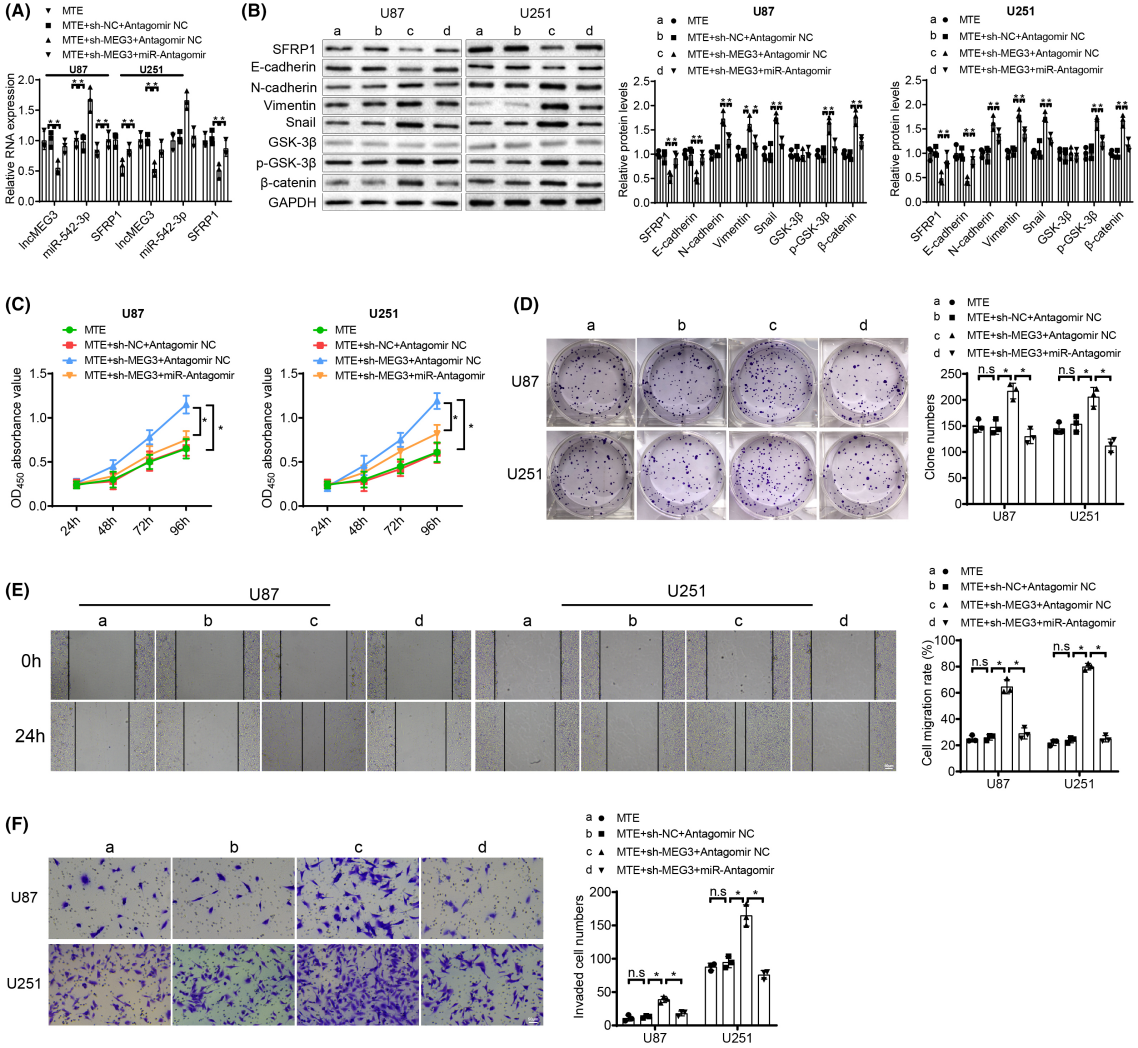
MTE upregulates SFRP1 and blocks the Wnt/β‐catenin pathway activation via the lncMEG3/miR‐542‐3p axis, thereby restricting glioma cell malignant behaviors. U87 and U251 cells in response to MTE treatment were then transfected with lncMEG3 shRNA and miR‐542‐3p Antagomir: A. LncMEG3, miR‐542‐3p and SFRP1 expression was determined by qRT‐PCR. B. The Wnt/β‐catenin pathway‐associated factors (GSK‐3β, p‐GSK‐3β and β‐catenin) and EMT‐related factors (E‐cadherin, N‐cadherin, Vimentin and Snail) were tested by western blot analysis. C. Cell viability was analyzed by CCK‐8 assay. D. Cell proliferation ability was determined by colony formation assay. E. Cell migration ability was detected by wound healing assay. F. Cell invasion ability was tested by Transwell assay. n.s refers to no significance; *p < 0.05. *N* = 3.

Lastly, we probed whether the MTE‐mediated inhibition of glioma cells worked by suppressing the Wnt/β‐catenin pathway activation and elevating SFRP1 expression via the lncMEG3/miR‐542‐3p axis. U87 and U251 cells upon MTE treatment were then transfected with lncMEG3 shRNA and miR‐542‐3p Antagomir. Results suggested that there were decreased lncMEG3, SFRP1 and E‐cadherin expression levels while increased miR‐542‐3p, p‐GSK‐3β, β‐catenin, N‐cadherin, Vimentin and Snail expression in the MTE + sh‐MEG3 + Antagomir NC group in contrast to the MTE + sh‐NC + Antagomir NC group; there were enhancements in the expression levels of lncMEG3, SFRP1 and E‐cadherin and reductions in the expression levels of miR‐542‐3p, p‐GSK‐3β, β‐catenin, N‐cadherin, Vimentin and Snail in the MTE + sh‐MEG3 + miR‐Antagomir group versus the MTE + sh‐MEG3 + Antagomir NC group (Figure 8A,B). Furthermore, the cell function experimentations of U87 and U251 cells uncovered that the capacities for glioma cells to proliferate, migrate and invade were strengthened in the MTE + sh‐MEG3 + Antagomir NC group in comparison to the MTE + sh‐NC + Antagomir NC group; the capacities for glioma cells to proliferate, migrate and invade were weakened in the MTE + sh‐MEG3 + miR‐Antagomir group versus the MTE + sh‐MEG3 + Antagomir NC group (Figure 8C–F). To sum up, MTE impedes the malignant behaviors of glioma cells via inhibiting miR‐542‐3p, upregulating SFRP1 and blocking the Wnt/β‐catenin pathway. The above results imply that the MTE‐induced inhibition of glioma cells was realized through upregulation of SFRP1 by the lncMEG3/miR‐542‐3p axis, and the inhibition of the Wnt/β‐catenin pathway activation.

### 3.7 MTE inhibits in‐situ glioma formation in nude mice

**FIGURE 9:**
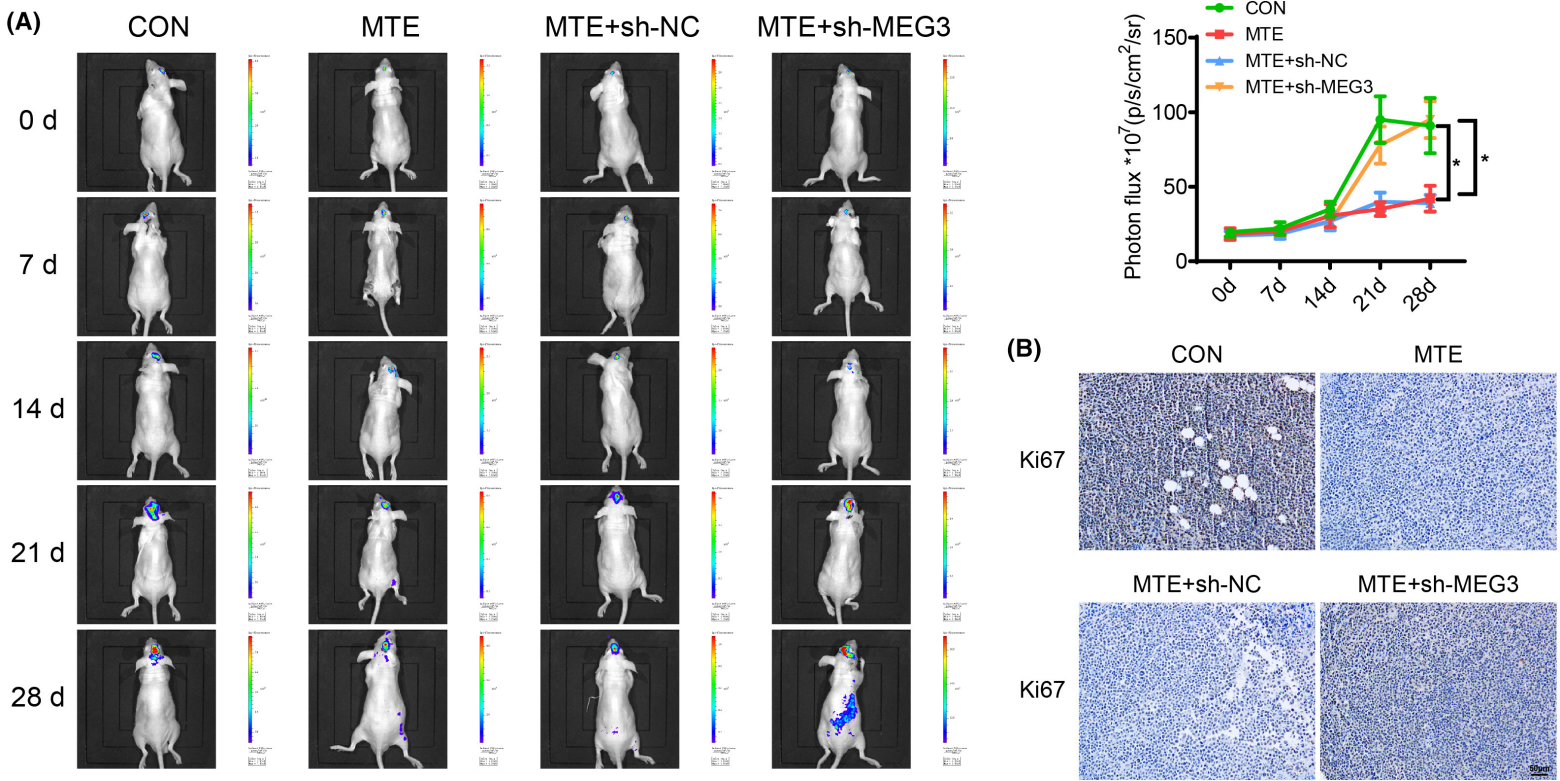
MTE inhibits in‐situ glioma formation in nude mice. A. In vivo imaging for observing the bioluminescence intensity in brains of nude mice. B. Ki‐67 immunohistochemistry was implemented on the brain tumor tissues in the nude mice. *p < 0.05. *n* = 6.

**FIGURE 10:**
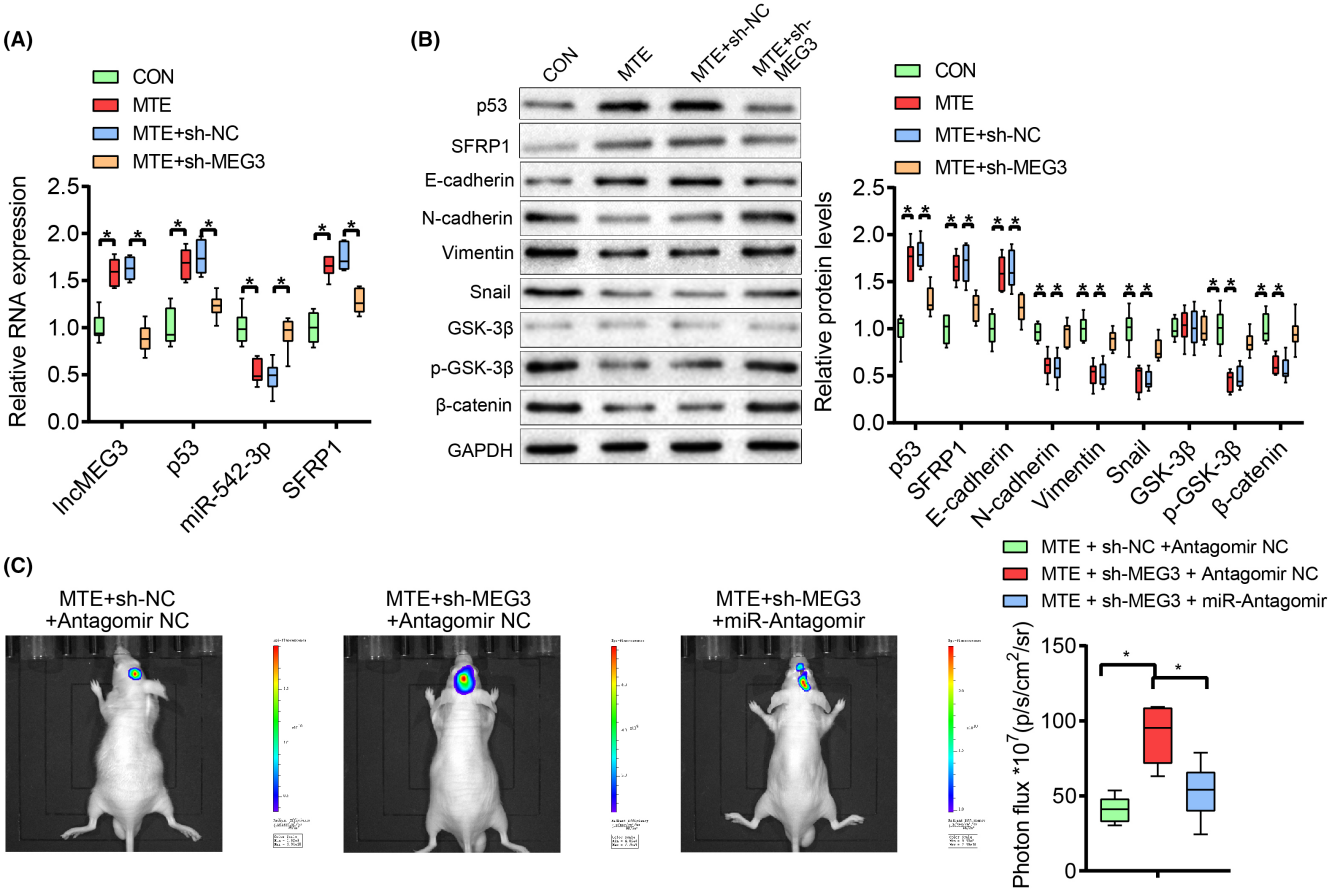
MTE inhibits in‐situ glioma formation via lncMEG3. A. LncMEG3, p53, miR‐542‐3p, and SFRP1 expression in the transplanted tumors was determined by qRT‐PCR. B. The p53 protein, SFRP1 protein, Wnt/β‐catenin pathway‐associated factors (GSK‐3β, p‐GSK‐3β, and β‐catenin), and EMT‐related factors (E‐cadherin, N‐cadherin, Vimentin, and Snail) were tested by western blot analysis. C. In vivo imaging for observing the bioluminescence intensity in brains of nude mice. *p < 0.05. *n* = 6.

**FIGURE 11:**
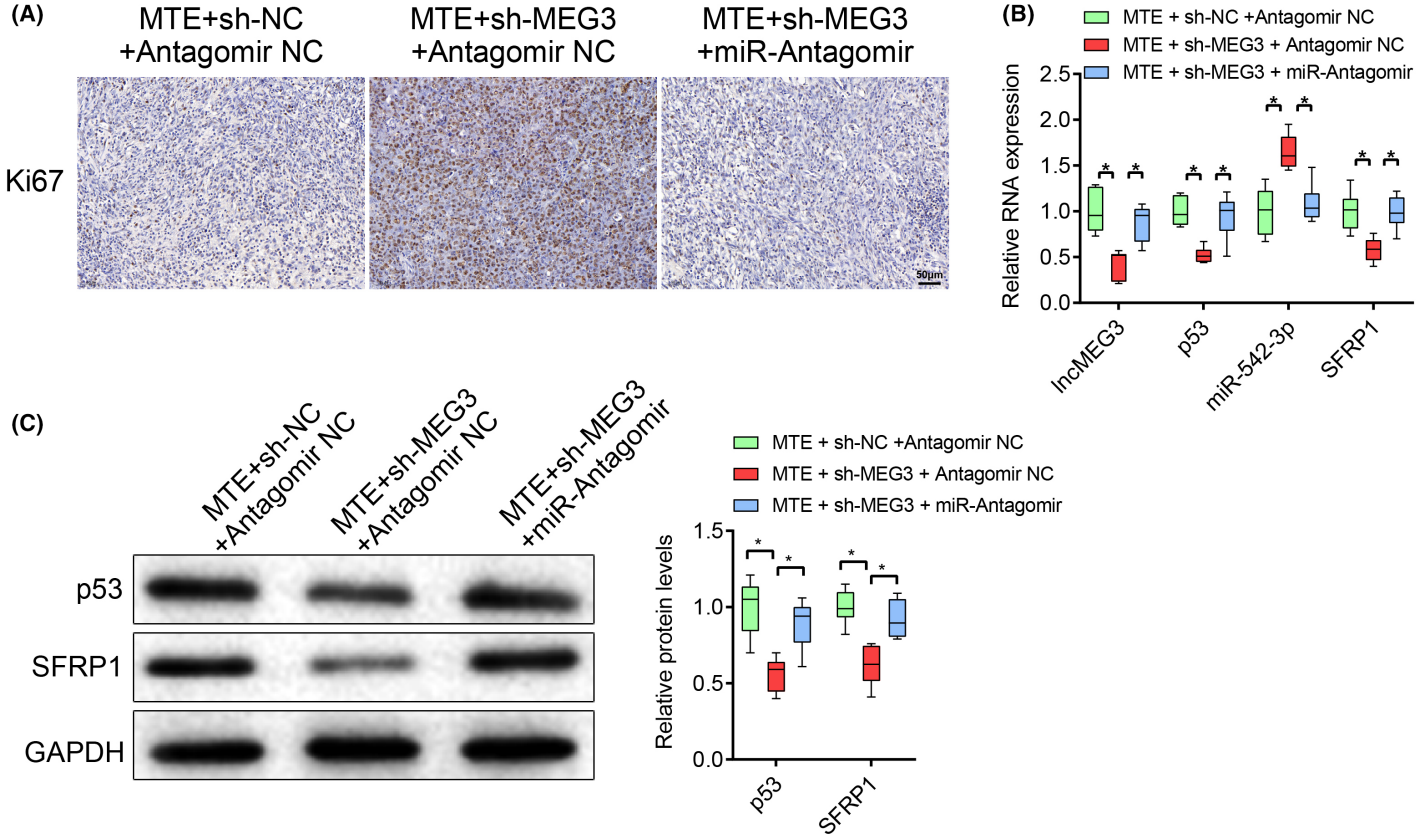
MTE inhibits in‐situ glioma formation via lncMEG3/miR‐542‐3p/SFRP1. A. Ki‐67 immunohistochemistry was implemented on the brain tumor tissues in the nude mice. B. LncMEG3, p53, miR‐542‐3p, and SFRP1 expression in the transplanted tumors was determined by qRT‐PCR. C. The protein levels of p53 and SFRP1 were determined by western blot analysis. *p < 0.05. *n* = 6.

At the animal level, we finally verified the effect of MTE on glioma in situ in nude mice. The nude mice were treated with lncMEG3 shRNA‐ or NC shRNA‐transfected U251‐GFP‐Luc cells by transplantation, followed by the intraperitoneal injection of 200 μg/mL MTE. The in vivo imaging of nude mice manifested that at 0, 7, and 14 days after modeling, the four groups of nude mice all presented luminescence in vivo, without significant difference in the luminescence intensity. At 21 and 28 days after modeling, there were enhanced bioluminescence intensity of mice in all the four groups; relatively weakened bioluminescence intensity was observed in mice with MTE treatment, which was enhanced in mice treated with both MTE and sh‐MEG3 (Figure 9A). Next, Ki‐67 immunohistochemistry was implemented on the brain tumor tissues in the nude mice, which addressed that the number of Ki‐67 positive cells was reduced in mice upon MTE treatment, while the number of which was increased in mice treated with both MTE and sh‐MEG3 (Figure 9B). Furthermore, there were elevated expression levels of lncMEG3, p53, SFRP1, and E‐cadherin, and downregulated expression levels of miR‐542‐3p, p‐GSK‐3β, β‐catenin, N‐cadherin, Vimentin and Snail in tumor tissues of mice upon MTE treatment, and the tendencies were reversed in mice treated with both MTE and sh‐MEG3 (Figure 10A,B). On day 28 after modeling, there were reductions in the bioluminescence in mice treated with MTE, sh‐MEG3, and miR‐542‐3p Antagomir relative to those treated with MTE, sh‐MEG3, and Antagomir NC (Figure 10C). The effects of sh‐MEG3 transfection on Ki‐67, lncMEG3, p53, SFRP1, and miR‐542‐3p expression were reversed by miR‐542‐3p Antagomir transfection in MTE‐treated mice (Figure 11A–C). The aforesaid findings disclose that MTE can inhibit the growth of glioma in situ in nude mice via the lncMEG3/miR‐542‐3p‐mediated upregulation of SFRP1 and suppression of the Wnt/β‐catenin pathway.

## 4 DISCUSSION

As a TCM herb, Marsdenia tenacissima not only impairs the growth of lung cancer cells (A549 and LLC) and motivate their apoptosis but also shows an antitumor activity in vivo.^28^ Its extract MTE also exerts a potent potential of inducing apoptosis through upregulating proapoptotic Bax, caspase‐9, and caspase‐3 and downregulating cyclin D1 and antiapoptotic Bcl‐2 in hematologic neoplasm cells.^10^ Also, MTE can attenuate ovarian cancer cell (SKOV3) growth and their resistance to apoptosis.^11^ Given the antitumor effect of MTE in these cancers, we aimed at assessing its function in glioma. Our main findings evidenced that 100 mg/mL and 200 mg/mL of MTE appreciably impeded the in vitro growth, colony‐forming, migratory, and invasive functions as well as in vivo oncogenicity of glioma cells (U87 and U251), accompanied with reversal of the EMT process. Furthermore, we probed into the possible mechanism by which MTE exerted its antitumor impact on glioma cells.

First of all, our study unveiled that lncMEG3 could be elevated by MTE in the glioma cells. LncMEG3 is regarded as an underexpressed lncRNA in glioma tissues/cells while reexpression of lncMEG3 leads to impaired proliferation, migration, and invasiveness in glioma cells (GSC11 and D54).^29^ A study has illustrated that deficiency of lncMEG3 shares a strong association with advanced WHO grade of glioma, tumor relapse, short overall survival, etc.^30^ Reversely, enhancement of its expression considerably restrains the glioma cell proliferation while stimulating their apoptosis and autophagy.^30^ Another study further demonstrates that lncMEG3 knockdown can augment glioma U118 cell growth through binding to miR‐377.^31^ In line with the aforementioned findings, our study offered in vitro and in vivo evidence substantiating that MTE‐induced elevation of lncMEG3 could prevent the malignant growth of glioma cells. Cell and animal experiments additionally confirmed that lncMEG3 knockdown significantly attenuated the tumor‐suppressive action of MTE, rendering lncMEG3 elevation as a downstream mechanism for the antitumor capacity of MTE in glioma.

Our subsequent mechanistic investigation suggested that lncMEG3 and SFRP1 competitively bind to miR‐542‐3p. Although miR‐542‐3p has been characterized to be one of the top upregulated miRNAs in GBM based on the intersection results of three microarray datasets, namely GSE25631, GSE42657, and GSE61710,^32^ its functions in glioma remains largely unknown. Intriguingly, miR‐542‐3p agomir was unveiled to enhance the malignant properties of glioma cells in the presence of MTE, suggestive its tumor‐promotive role in glioma. Inhibition of miR‐542‐3p in MTE‐treated mice abrogated lncMEG3 silencing‐facilitated in‐situ tumor growth. Following the previous finding,^33^ SFRP1 was demonstrated as a target gene of miR‐542‐3p from our results of dual‐luciferase reporter assay. Additionally, SFRP1 overexpression was witnessed to repress the proliferative, migratory, and invasive functions of glioma cells as well as to block the induced EMT during cancer progression.

Lastly, SFRP1 was unveiled to block the Wnt/β‐catenin pathway, whereby limiting the malignant behaviors of glioma cells. SFRP1‐dependent inhibition of the Wnt/β‐catenin pathway has been expounded to hinder the tumorigenesis and tumor metastasis in several cancers such as epithelial ovarian cancer^34^ and nasopharyngeal carcinoma.^35^ Coincidentally, several studies have additionally revealed the involvement of SFRP1/Wnt in glioma. For instance, elevated SFRP1 protein levels in glioma patients are linked to prolonged overall survival; downregulation of SFRP1 and activation of Wnt may give rise to the development of infiltrative glioma phenotype during the early phrases of glioma progression.^36^ Three SFRP members including SFRP1 can reduce Wnt signaling and thus impede proliferative activity and anchorage‐independent growth of medulloblastoma cells; meanwhile, SFRP1 curbs in vivo formation of medulloblastoma.^37^ SFRP1 also impairs the malignant growth of glioma in vitro, which is indicated to be potentially linked to the blockade of the Wnt/β‐catenin pathway.^21^ Recombinant SFRP1 acts as an inhibitor of nuclear β‐catenin in glioma stem cells, halting their proliferation and inducing apoptosis.^38^ A more recent study has unveiled that Wnt signaling can be activated in GBM when SFRP1 is epigenetically silenced.^39^ Our results supplied additional evidence that miR‐542‐3p‐induced inhibition of SFRP1 and resultant SFRP1‐dependent blockade of the Wnt/β‐catenin pathway underpinned the tumor‐inhibitory effect of lncMEG3 elevation in glioma following MTE treatment.

All in all, this study unraveled that MTE treatment contributed to prevention against the growth and progression of glioma, highlighting its promise as a potential alternative therapy for glioma patients. Exploration on the molecular mechanisms suggests that activation of the lncMEG3/miR‐542‐3p/SFRP1 axis contributed to the antitumor action of MTE, which gives insight into the crucial significance of lncRNA/miRNA/mRNA and pathways in the TCM treatment of patients with cancers. Neovascularization is a major factor in tumor progression and metastasis. More future in vivo studies are planned to investigate the MTE treatment of brain glioma in respect of tumor angiogenesis. Molecular magnetic resonance imaging^40^ and quantitative ultramicroscopy^41^ can be used to analyze various parameters of vessel structures (i.e., vessel length, radius, tortuosity, etc.) to characterize in‐situ glioma formation and its response to MTE. Thus far, clinical delivery of the majority of antiglioma drugs is restricted at the blood–brain barrier by ABCB1 and ABCG2.^42^ A previous study has shown that intraperitoneal administration of MTE can suppress the activity of ABCG2 in lung cancer xenografts.^43^ Moreover, polyoxypregnane compounds isolated from MTE have been reported to overcome ABCB1‐ or ABCG2‐mediated multidrug resistance in cancer cells.^44^, ^45^ Therefore, the efficiency of MTE brain delivery may not be restricted by ABCB1 and ABCG2, since itself can suppress the efflux activities of ABCB1 and ABCG2. The use of MTE alone or its use in combinational chemotherapy is worth more in vivo studies to improve the treatment of glioma.

## AUTHOR CONTRIBUTIONS

CL and HMY conceived the ideas. CL and HMY designed the experiments. CL and GX performed the experiments. CL and GX analyzed the data. CL and GX provided critical materials. CL and GX wrote the manuscript. HMY supervised the study. All the authors have read and approved the final version for publication.

## FUNDING INFORMATION

This research was funded by the grants from Scientific Research Planning Program of Health and Family Planning Commission of Hunan Province (Grant No. C202104041346) and Chinese Medicine Scientific Research Planning Project of Hunan Province in 2021 (Grant No. 2021207).

## CONFLICT OF INTEREST STATEMENT

The authors have nothing to be declared.

## Data Availability

The datasets used or analyzed during the current study are available from the corresponding author on reasonable request.
